# A roadmap to understanding interoceptive awareness and post-traumatic stress disorder: a scoping review

**DOI:** 10.3389/fpsyt.2024.1355442

**Published:** 2024-05-22

**Authors:** Kelly Leech, Peta Stapleton, Alan Patching

**Affiliations:** School of Psychology, Bond University, Gold Coast, QLD, Australia

**Keywords:** interoceptive awareness, post-traumatic stress disorder, scoping review, trauma, emotion regulation

## Abstract

The concept of interoception has existed since the beginning of the 1900s. It is suggested that humans can observe feelings arising from the body that allows them to develop a sense of their emotional status and physical condition. The term interoceptive awareness appears to originate with clinicians working with individuals who had experienced trauma, in particular Post Traumatic Stress Disorder (PTSD). The purpose of this scoping review was to provide an overview of the existing literature surrounding the combination of these two themes: interoceptive awareness and PTSD. A total of 226 articles were initially screened and 52 articles were retained for comprehensive review. Nine articles were excluded, resulting in 43 studies included in the review. The review aimed to answer: (a) how is interoceptive awareness defined? (b) how is interoceptive awareness measured? (c) what is the function of interoceptive awareness? (d) is there/what is the relationship between interoceptive awareness and PTSD? The scoping review identified nine terms that are used synonymously throughout the literature surrounding interoceptive awareness and PTSD, and three primary ways in which interoceptive awareness is measured in relation to PTSD. The primary function documented was the role interoceptive awareness played in an individual’s ability to regulate their emotions, and the most common and compelling function emerging was the association with emotion regulation. The evidence supports the utilisation of a definition of interoceptive awareness to include one that includes the quality of cognitive appraisal and focuses on the adaptive mindful approach to internal physical sensations as opposed to the heightened ruminative self-focus. Limitations and future research are suggested.

## Introduction

Sherrington (1) first raised the idea that an individual has the ability to receive sensory input internally from the body. However, interoception and derivative terms thereof have been of more clinical and research focus since the turn of this century. In a model of psychosomatic processes, interoception denotes an individual’s ability to consciously recognise internal bodily changes, signals, or cues (2). It has been suggested that there are three stages involved in interoceptive processing. As proposed by Schulz and VöCheck that all equations and special characters are displayed correctly.gele (3) these include: firstly, signals originating in the viscera are sent to the brain; secondly, awareness and attention is subsequently directed towards these signals, and thirdly an appraisal of the signals occurs for their individual meaning. Consensus across the literature supports a general definition of interoceptive awareness to incorporate a perception of the internal state of the body (4). Defining interoceptive awareness, measuring it, and understanding how it interplays with aspects of an individual’s well-being is complex and momentum is building in the research to better conceptualise this subject. To improve understanding on this emerging topic, an overview of the literature was conducted.

**Figure 1:**
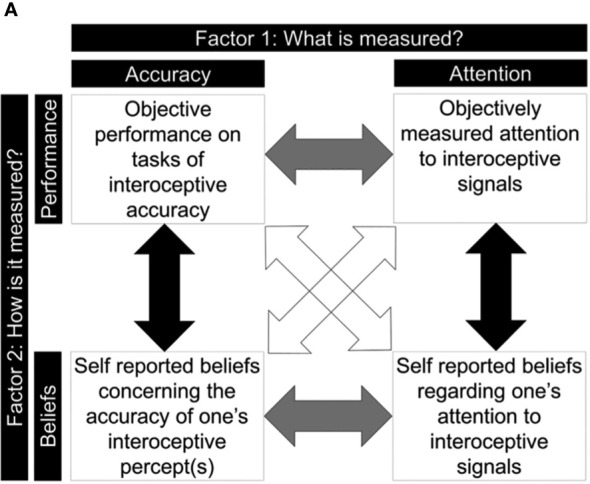
A 2 x 2 Factorial Model of Interoceptive Ability (6).

As highlighted, research on interoception is increasing with more specific construct definitions beginning to emerge. In a three-dimensional model outlined by Garfinkle et al (5), distinctions have been proposed between interoceptive accuracy, interoceptive sensibility, and interoceptive awareness. Interoceptive accuracy is defined as the ability to objectively identify internal body signals, interoceptive sensibility is defined as the self-perceived ability to recognise internal body signals, and lastly, interoceptive awareness is defined as the ability to be aware of how accurate individuals are at detecting their internal body signals (5). The modes of assessment for each term are proposed to consist primarily of objective tests for interoceptive accuracy such as the heartbeat detection tasks, and subjective tests for interoceptive sensibility such as questionnaires (5). Interoceptive awareness, as defined in the three-dimensional model, is measured by the relationship between objective performance and perceived performance (5). A 2 x 2 factorial model has subsequently been proposed expanding on the three-dimensional model (**Figure 1**; 6). This distinguishes between two main domains in interoception, target of measurement: accuracy versus attention, and type of measurement: objective performance versus self-reported beliefs (6). Despite more specific definitions of terminology around interoceptive awareness emerging, there is a lack of consistency across fields of research.

### Theoretical underpinnings of interoceptive awareness

Interoceptive awareness is an important concept in the field of psychology with theoretical and research underpinnings stemming from outcomes originally observed by Ivan Pavlov (7). Classical and operant conditioning directed the focus on understanding the theoretical foundation of interoceptive conditioning (8). Pavlov recognised that the body’s internal environment can be influenced by events and can in turn influence behaviours (7). Interoceptive conditioning defines the style of conditioning whereby information regarding the state of the body forms either the unconditioned or conditioned stimulus (9). Interoceptive conditioning is suggested to contribute to a range of pathologies (10) and is proposed to be significant in the cause and maintenance of mental health disorders such as mood and anxiety disorders (11) and eating disorders (12). Paulus et al (13) have extended this theory in modern society to suggest that interoceptive awareness is involved in motivational processes, and dysfunctional or misinterpreted interoceptive awareness can contribute to maladaptive behaviours. Interoceptive conditioning aids understanding of how internal body sensations can be influenced by environmental factors, and dysfunction in the interpretation of these sensations is a potential contributor to mental health disorders.

### Function of interoceptive awareness

Early proposals of the function of interoceptive awareness suggest that a healthy embodiment is fundamental in experiencing a sense of centeredness and grounding as well as security and pleasure in the body (14). Interoceptive awareness has been found to be beneficial in the health and well-being of an individual, as it provides the gateway to responding effectively to visceral changes in order to maintain homeostasis (15). Without accurate interoceptive awareness, regulation of internal bodily changes becomes more challenging (5). A link has been found between interoceptive awareness of physical sensations associated with emotions and the ability to regulate affect and engage in effective decision-making (16, 17). Research has supported this connection between interoceptive awareness and affect regulation, indicating that poorer interoceptive awareness is present in individuals experiencing emotional disorders (11). Additionally, higher interoceptive awareness has been identified to enable the downregulation of emotions and individuals’ ability to perceive their internal bodily cues determines their emotion regulation ability (18). The connection between interoceptive awareness and effective decision-making is theoretically founded on Damasio’s Somatic Marker, and neuroscientific findings have established that activity in the right anterior insula, an area involved in the interoceptive processing, was related to decision-making performance (19). Further to this, empirical research has supported this association finding that depressed females experiencing poorer interoceptive accuracy were associated with difficulty in decision making (20).

### Interoceptive awareness and PTSD

The term interoceptive awareness in relation to the understanding and treatment of mental health disorders, specifically PTSD, appeared to originate with clinicians working with individuals who had experienced trauma. Van der Kolk (21) stated that through his clinical experience, individuals with PTSD have trouble attending to internal sensations as they commonly become overwhelmed due to residual trauma-related perceptions, emotions, and sensations (22). Through evidence of the impact of trauma on the functioning of the brain, Van der Kolk (21) advocated for the focus of treatment to be on developing individuals’ interoceptive capacity in the treatment of PTSD in order to improve their ability to tolerate feelings and sensations. Similarly, somatic experiencing, developed by Levine (1977), Levine (1997), Levine, 2010) (23–25) is a form of therapy which focuses on treating symptoms of post-traumatic stress. Somatic experiencing advocates for bottom-up processing whereby the focus is specifically on directing attention to a client’s internal world, both interoception and proprioception, as opposed to utilising a top-down cognitive approach (26). There is compelling evidence for the benefits of a well-developed interoceptive awareness, but for many individuals experiencing PTSD, interoceptive awareness can be compromised (25). Support for this approach is bolstered through the benefits observed in sensorimotor psychotherapy (27), whereby interventions for individuals who have experienced trauma focus on somatic awareness, including mindfulness of interoceptive states and building body-oriented resources to regulate arousal. Examples of somatic techniques utilised within sensorimotor psychotherapy include mindful postural practices and pendulation with uncomfortable emotions and bodily sensations. With empirical data displaying a link between interoceptive awareness and emotion regulation and decision-making, as well as individuals presenting for treatment of PTSD commonly displaying deficits in interoceptive awareness, further understanding and research into this area is justified.

With the diversity of information available on the term interoceptive awareness it is argued that undertaking a scoping review exploring how specifically interoceptive awareness has been investigated in relation to the mental health disorder, PTSD, is important prior to undertaking further research in this field. The purpose of this scoping review is to provide an overview of the existing literature surrounding the combination of two themes: interoceptive awareness and PTSD. A scoping review has been selected over a systematic literature review, despite both being a type of evidence synthesis. This was due to the nature of the research questions and the required outcome. It is reported that scoping reviews are specifically beneficial when needing to identify knowledge gaps in a particular field, to clarify concepts and definitions, examine how research has been conducted and whether certain factors are related to a particular concept (28). The outcome of this scoping review is proposed to inform and guide subsequent studies. This scoping review has three specific objectives: conduct a systematic search for published papers on interoceptive awareness and PTSD, outline the existing data pertaining to specific research questions, and to identify research gaps in the existing literature and propose future research projects to enhance investigation in this area.

## Method

This scoping review adopted a methodological framework outlined by Arksey and O’Malley (29) and included the following areas: identification of research questions; searching and selection of relevant studies; mapping of the data; and a summarisation of the results.

### Research questions

This scoping review was guided by the following research questions. Within the research pertaining to interoceptive awareness and PTSD:

- How is interoceptive awareness defined?
- How is interoceptive awareness measured?
- What is the function of interoceptive awareness?
- What is the relationship between interoceptive awareness and PTSD?

### Data sources and search strategy

The final search was completed in January 2022, focusing on published journal articles and texts in the years proceeding which related to both interoceptive awareness and PTSD, including common derivatives of both terms. Three databases were selected to cover a broad range of relevant disciplines and included Psychinfo, Pubmed, and EBSCO, as well as the first two pages of google scholar to ensure any missed texts were collated and reviewed. Terms used in the search query included: interoception, interoceptive awareness, interoceptive sensibility, interoceptive accuracy, body awareness, somatic awareness, PTSD, post-traumatic stress, post-traumatic stress disorder.

### Data screening and summary

**Figure 2:**
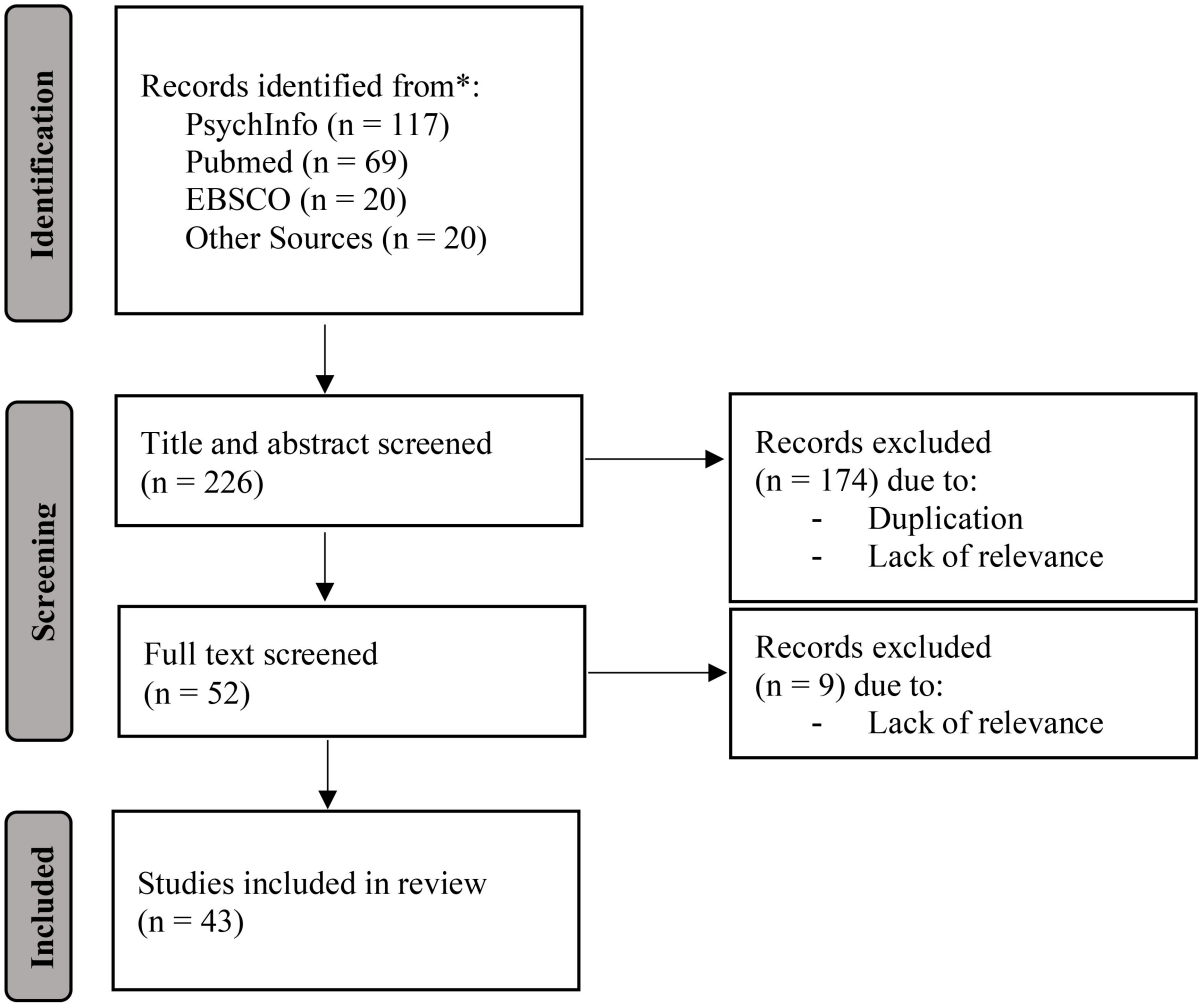
A Flow Diagram Depicting the Screening Process of the 43 Studies Within this Scoping Review.

Each article returned using the search queries was screened initially through review of the title and abstract for key terms. A total of 226 articles were screened using this initial strategy. Articles were excluded if they did not refer to interoceptive awareness or PTSD or a derivative of those terms (see above) or were duplicates of one another. Additionally, due to limitations in resources available for translation, only articles published in English were selected. Furthermore, articles which solely reflected a medical investigation with no inclusion of an examination of psychological constructs were excluded due to lack of relevance. Articles which were considered to be relevant post review of the title and abstract were retained on an excel database for full article analysis. A total of 52 articles were retained for comprehensive review. Upon review of the full text, nine articles were not included in the review due to lack of relevance to the research questions (see **Figure 2** for a CONSORT Prisma flowchart of the data screening process). Information from articles was utilised both quantitatively and qualitatively to provide a comprehensive understanding of the literature available in relation to the combination of the two concepts, interoceptive awareness and PTSD.

Results

**Figure 3:**
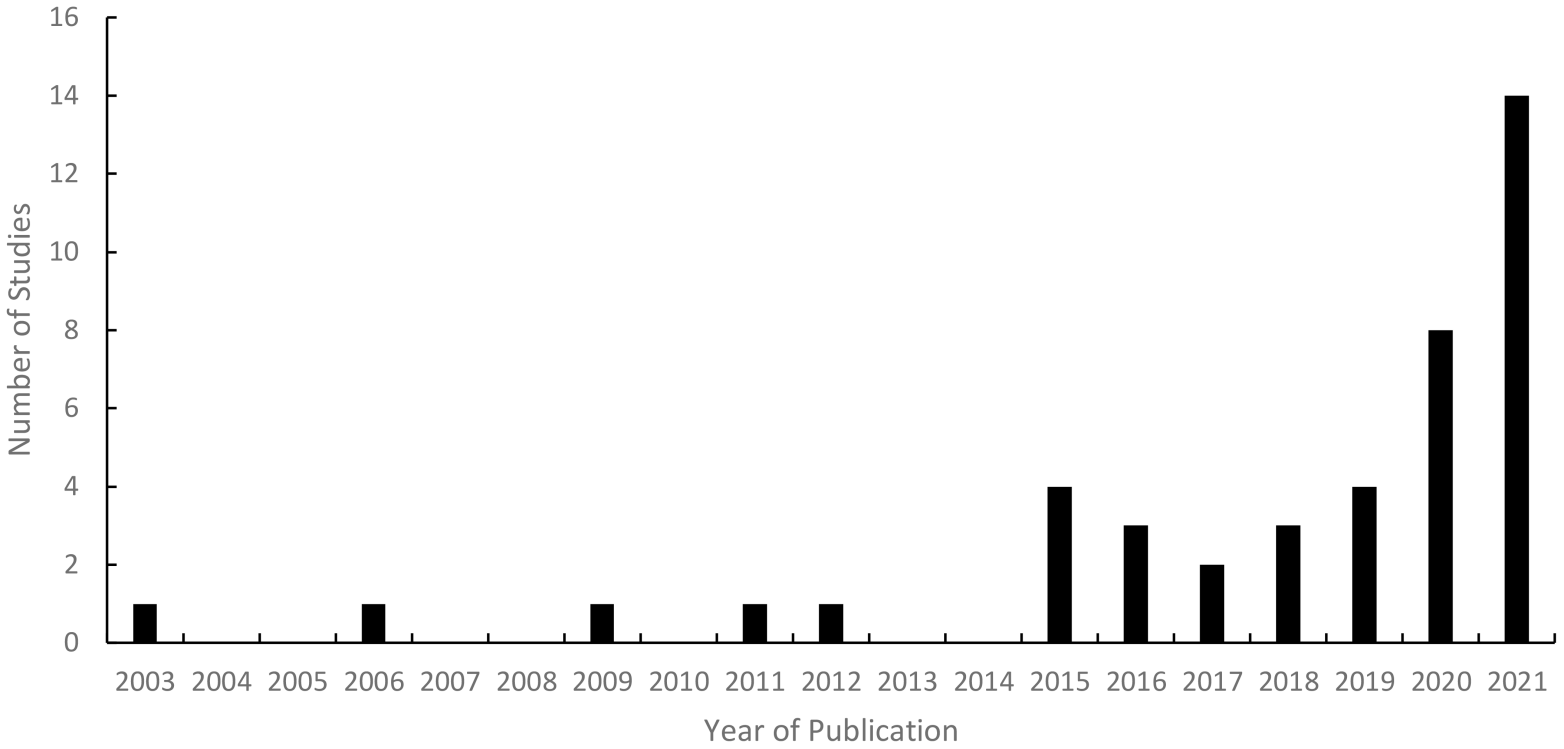
Number of Studies Published Each Year on Interoceptive Awareness and PTSD.

Prior to reviewing the 43 studies included in this scoping review in detail, a notable trend emerged in the collated data around the increasing occurrence of research conducted into interoceptive awareness and PTSD in recent years. The data displayed a significant upturn in studies published in this field from 2015 onwards, with less than 12% of the studies deemed relevant for this scoping review published in the years prior to 2015. Markedly, there was a 43% increase in studies conducted pertinent to this topic from 2020 to 2021 (see **Figure 3** for a bar chart of the data distribution of journals published over the years).

### Defining interoceptive awareness

Numerous definitions have been used to understand interoceptive awareness, largely due to the lack of consistency in terminology used across disciplines. The scoping review identified nine terms to be used synonymously throughout the literature surrounding interoceptive awareness and PTSD: these included interoception, interoceptive awareness, interoceptive accuracy, interoceptive sensitivity, interoceptive sensibility, interoceptive processing, interoceptive deficits, body awareness, and somatic awareness. Multiple terms have also appeared to be utilised interchangeably within the same journal article. The most commonly used terms identified in the selected studies was interoception and interoceptive awareness. Amongst this literature, a definition which repeatedly emerges is that offered by Craig (30). Craig (30) outlines interoception as the ability to observe and interpret physical sensations that arise in the body which provides an individual with a sense of their physical condition and emotional state. Additionally, a widely recognised definition which is prominent in recent psychological research surrounding interoceptive awareness as a treatment outcome has focused on that proposed by Mehling et al. (31), which incorporates the critical component of physical sensation appraisal. For the purpose of this scoping review, particular attention will be paid to the definition proposed by Mehling et al. (31).

Wolf Mehling developed this understanding and definition of interoceptive awareness through research on the term, body awareness. He identified that the research indicated that the term body awareness, was historically associated with examining the heightened awareness and amplified focus on the internal physiological symptoms commonly seen in anxiety-related disorders such as panic disorder (31). This led to the presumption that body awareness was largely maladaptive. Despite this, many clinical interventions aim to improve and develop individuals’ body awareness as a clinical tool, useful for well-being. Mindfulness characterises the specific kind of body awareness which is deemed beneficial for individuals’ well-being, which comprises of non-judgemental awareness, acceptance, and a sense of embodiment (31). Focusing attention on present-moment emotions appears to be adaptive as opposed to a maladaptive ruminative self-focus. Research and definitions in the past on interoceptive awareness have largely steered clear of the key cognitive aspects of appraisal and beliefs, attention regulation, and behavioural aspects such as avoidance (31). Therefore, a broader conceptualisation of interoceptive awareness was suggested such as those proposed by Cameron (2) and Mehling et al. (31) which takes into consideration appraisal and interpretation of physical sensations.

### Measuring interoceptive awareness

There appears to be little consensus between disciplines in terms of a classification framework and definition of interoception to date, causing interoceptive awareness being measured using a variety of different methods. This scoping review elicited three primary ways in which interoceptive awareness is measured in relation to the published literature on interoceptive awareness and PTSD. In a large proportion of the studies which measured the concept of interoceptive awareness in individuals, a self-report measure was utilised, assessing individuals’ subjective awareness of internal bodily symptoms. Fewer studies in the scoping review utilised functional magnetic resonance imaging to investigate areas of the brain commonly associated with interoception. Lastly, one study investigated the use of an objective measure such as the heart beat perception task in comparison to a subjective measure of interoceptive awareness (32).

Based on the exploration of the definitions surrounding the term interoceptive awareness, the distinction between focus on interoceptive signals with a ruminative versus mindfulness approach is vital in determining the clinical utility of interoceptive awareness on PTSD. To understand how best to effectively measure this distinction, Mehling et al. (33) reviewed 39 self-report measures pertaining to body awareness and selected the 12 most relevant assessments to evaluate psychometrically. The outcome of this review revealed a neglect in assessing the critical mindfulness component of body awareness and the importance of developing a measure which distinguishes between the identified adaptive and maladaptive components of body awareness (33, 34). The multidimensional assessment of interoceptive awareness (MAIA) measure was developed as a result of this and is able to differentiate between an anxious and heightened ruminative focus on body sensations and a mindful and accepting awareness, which is clinically relevant in treatment (35). This measure was developed to assess interoceptive awareness as defined above by Mehling et al. (33). The MAIA or MAIA-2 was utilised or mentioned as an assessment tool for interoceptive awareness ten times (35–40; Norbrandt et al., 2020; 32, 34, 41) and appeared to be the most consistent psychological assessment measure used to assess an individual’s interoceptive awareness in the literature relating to interoceptive awareness and PTSD.

Neuroimaging studies are evident in the literature of interoceptive awareness and PTSD as being another source of measuring interoceptive awareness (42–44). Assessing the functioning of identified neural networks of the brain proposed to be involved in interoceptive awareness appear to provide insight into what affects an individual’s interoceptive awareness (e.g. trauma) and the functioning of these networks in individuals experiencing PTSD. Common areas of the brain of focus using functional magnetic resonance imaging (fMRI) in the literature appear to be the anterior, mid, and posterior insula region, amygdala, and dorsal anterior cingulate cortex (42, 44, 45). As many of the outcomes of these neuroimaging studies go beyond the purpose of this scoping review, details of the results are not discussed.

The heartbeat perception task, an objective assessment measuring interoception, asks participants to count their heartbeats over a select period of time. Their accuracy is assessed by calculating the difference between actual heartbeats and counted heartbeats. This mode of assessment has been thought to best measure interoceptive accuracy, the accuracy in perceiving internal body sensations, which has been shown theoretically and empirically to be a different construct to interoceptive sensibility. This is a person’s subjective awareness of inner body sensations (5, 46, 47). The lack of correlation between these modes of assessment is indicated in trauma survivors through a study which showed that outcomes from the heartbeat detection task did not correlate with the self-reported interoceptive sensibility measured with the MAIA-2 (32).

### Function of interoceptive awareness

The most common and compelling function emerging from the literature investigated in this scoping review on interoceptive awareness and PTSD is the association with emotion regulation. It is proposed that a function of interoceptive awareness is focussed on enhancing an individual’s emotion regulation ability. Weng et al. (48) identified mindfulness practice to enhance interoceptive awareness through interventions that focus attention on the inner sensations of the body with an open and curious mindset. They postulated that this would reduce reactivity to physical symptoms and through a more considered awareness of the importance of varying physical sensations, would improve emotion regulation (48). Their evidence for this centred primarily on the theoretical underpinnings of the Mindful Awareness in Body-Oriented Therapy (MABT) proposed by Price and Hooven (17), which presents a framework for the connection between interoceptive awareness and emotion regulation. Silverberg (34) utilised neuroscientific findings on the functioning and structure of the anterior insula and consequential representation of physical and emotional conditions (31, 49, 50) to draw the conclusion on the relationship between interoceptive awareness and affect regulation. Additionally, investigation into eating disorder symptomology offered support for the relationship between observed affect regulation difficulties and interoceptive difficulties (51). Despite the association between interoceptive awareness and emotion regulation being implicated numerous times in the literature, to the first author’s knowledge, this relationship has not been directly empirically investigated in individuals with PTSD.

The behavioural intervention, interoceptive exposure, provides further insight into the potential function of interoceptive awareness. Interoceptive exposure aims to increase an individual’s tolerance for their somatic sensations by reducing the distress that is related to the physical sensations (52). Systematic desensitisation to aversive physical sensations is suggested to be the mechanism behind interoceptive exposure in anxiety disorders as first revealed by Wolpe (53). It is proposed that during interoceptive exposure, an individual’s interoceptive sensitivity is altered (52), which provides an understanding into the benefit of this intervention for individuals with anxiety disorders.

### Interoceptive awareness and PTSD

This scoping review revealed many sources of evidence that supported the existence of the relationship between interoceptive awareness and PTSD as well as the nature of this relationship. Studies predominantly involving neuroimaging and clinical intervention trials emerged during the scoping review as being particularly beneficial in assisting our understanding of how interoceptive awareness and PTSD relate.

Neuroimaging studies on individuals with PTSD aid understanding into how trauma relates to an impairment in interoceptive awareness through changes to the functioning of specific areas of the brain. Interoceptive processing is reported to be altered in individuals with PTSD (44, 54). Neuroimaging studies have consistently found decreased activation of the medial prefrontal cortex in people with PTSD (55, 56). This decreased activation is thought to impair extinction of the fear response and hindering inhibition over the limbic system (57). Consequentially, the stress response is not appropriately suppressed (22), which is proposed to contribute to the arousal dysregulation experienced in PTSD (58). Additionally, perception of physiological signals, interoceptive awareness, is reported to be represented in the anterior cingulate cortex (59), part of the medial prefrontal cortex which is indicated to be structurally smaller and functionally impaired in individuals with PTSD (56). Despite this finding indicating decreased activation in key areas of the interoceptive nervous system associated with hyperarousal, other reports of neuroimaging studies have demonstrated increased anterior insula activation in individuals with greater symptoms of PTSD, suggesting heightened levels of body awareness or interoception (60, 61). Lastly, the review of the literature highlighted a neuroimaging study by Simmons et al. (45), who investigated activation in the neural networks in individuals with and without PTSD who were expecting a change in interoceptive state. The authors found similar prefrontal cortex activation between the groups, however, differing right anterior insula activation in the PTSD group. They suggested that these findings indicate a cognitive awareness by individuals experiencing PTSD of an upcoming shift in affect, however, they are not able to regulate the emerging interoceptive outcome (45).

Dissociation, a symptom at times experienced alongside hyperarousal symptoms for individuals with PTSD, has been linked with impairments in interoception (62). Dissociation refers to a state of disconnection or altered engagement with one’s internal and external world (62). Notably, it can present as disconnection from one’s own body and therefore the internal physical sensations that occur. Outcomes of neuroimaging studies have further suggested that there is decreased activation of the insula, during times of hypo-arousal in individuals with PTSD, which is accompanied by diminished interoceptive awareness (61). Fifteen to 30 percent of individuals with PTSD were reported to exhibit emotional overmodulation, characterised by diminished interoceptive awareness (44).

#### Clinical intervention trials

In regards to clinical intervention trials, studies emerged in this scoping review involved the implementation of a treatment for PTSD which additionally assessed changes in interoceptive awareness. One study investigated a 12-week integrative exercise program which incorporated physical exercises with mindfulness practices which was provided to 47 war veterans diagnosed with PTSD (35). Outcomes of this intervention saw significant improvements in PTSD symptoms coupled with improvements in interoceptive awareness (35). Specifically, the multidimensional assessment of interoceptive awareness (MAIA) was used as a measure for interoceptive awareness and the specific elements of interoceptive awareness which demonstrated a significant increase was, body listening (listening to the body for insight) and self-regulation (ability to regulate psychological distress by paying attention to internal sensations) (35). As this was a limited pilot study, a mediation analysis was unable to be conducted to identify if changes in interoceptive awareness was the mediating factor in the relationship between the intervention and PTSD symptom change.

Similarly, a 16-week yoga program was provided to 209 individuals with PTSD and was compared against a wellness lifestyle program (39). This study also demonstrated a significant reduction in PTSD symptoms at the mid and end of treatment point for those in the yoga program compared to the wellness lifestyle program as well as in secondary outcomes such as the interoceptive awareness elements of attention regulation (ability to sustain and control attention to body sensations), body listening (listening to the body for insight), emotional awareness (awareness of the connection between body sensations and emotional stated), and self-regulation (ability to regulate psychological distress by paying attention to internal sensations) (39). As with the integrative exercise program, hypothesised mechanisms behind the improvement in PTSD symptoms involve elements of interoceptive awareness, however, no analysis was performed in this study to test this hypothesis (39).

Conversely, not all intervention studies displayed outcomes which supported the benefit of building interoceptive awareness for treatment of PTSD. When comparing the benefit of aerobic exercise with aerobic exercise and the addition of interoceptive prompts (whereby participants’ attention was directed to their somatic arousal elicited from the exercise), the investigation found a similar reduction in PTSD symptoms (63). The authors of this study noted this result as contradicting their original hypothesis and queried whether the outcome was as a result of all participants engaging in exercise no matter which group they were assigned, received implicit interoceptive exposure (63). Additionally, a study exploring the effectiveness of Internal Family Systems (IFS; 64) for adults with PTSD showed a significant reduction in PTSD symptoms after 16 sessions, but only a small significant change in one out of the eight elements of interoceptive awareness, as measured using the MAIA-2, non-distracting, the ability to refrain from using distraction or ignoring to cope with discomfort in the body (40). This result does not support the association between PTSD and interoceptive awareness, as when PTSD symptoms reduced, little change was observed in the elements of interoceptive awareness. However, as the two concepts were not directly assessed in relation to each other, it cannot be concluded that interoceptive awareness is not the mechanism behind PTSD symptom change.

An intervention which emerged several times in this scoping review as an intervention for PTSD, or an addition to already existing treatments, is Basic Body Awareness Training (BBAT). This was based on clinical experience displaying an increasing demand for interventions focusing on the physiological reactions to PTSD (65). The effect of BBAT on veterans with PTSD has been shown to potentially increase bodily awareness as well as qualitative reports suggesting a decrease in hyperarousal experiences (65). Additionally, BBAT has been suggested to compliment Cognitive Behavioural Therapy for treatment for PTSD as it provided a more specific bodily-oriented approach, which enhanced calmness and relaxation as well as improving awareness of signals arising in the body by shifting away from a pure cognitive focus (66). A study protocol has been proposed by Ahlmark, et al. (38) for a randomised controlled trial investigating body therapy as an adjunct to treatment as usual for veterans with PTSD. The authors outline their rationale that the intervention will improve interoceptive awareness in the short-term which will have a long-term impact on reducing PTSD (38). Lastly, the research revealed that the mechanisms behind the improvement in PTSD symptoms witnessed after application of a mindfulness intervention are proposed to be in part due to improved interoceptive awareness and emotion regulation (67). Mindfulness underpins the adaptive response to interoceptive cues through attending to and appraising physiological signals and is largely suggested to be where the advantage of mindfulness is derived from (68). Purely increasing attention to physiological symptoms has the potential to be detrimental for trauma survivors (4) and therefore it is deemed important to enable individuals to tolerate internal sensations without applying a maladaptive coping strategy (69).

Studies included in the scoping review also displayed support for a relationship between interoceptive awareness and PTSD through the examination of the concepts in individuals experiencing an eating disorder. Individuals experiencing an eating disorder have been associated with a greater risk for also experiencing PTSD (70). A study investigated the treatment effect of cognitive processing therapy on individuals with PTSD and eating disorders (43). The results of this displayed support for the notion that PTSD and an eating disorder together are partly due to shared factors such as emotion dysregulation, interoceptive awareness dysfunction, and impulsivity (43). Additionally, individuals who experienced an eating disorder (57 people with anorexia nervosa, 26 with bulimia nervosa, 18 with eating disorder not otherwise specified, and 29 with binge eating disorder) with PTSD (33.9% of the participants) were reported to display significantly higher impulsivity and lower interoceptive awareness amongst other variables compared to those individuals who experienced an eating disorder alone (70). These studies investigating participants with both an eating disorder and PTSD both support the possible relationship between PTSD and impaired interoceptive processing.

## Discussion

This scoping review aimed to conduct a systematic search for published papers on interoceptive awareness and PTSD and outline the existing data pertaining to the specific research questions, within the research pertaining to interoceptive awareness and PTSD: (a) how is interoceptive awareness defined? (b) how is interoceptive awareness measured? (c) what is the function of interoceptive awareness? (d) is there/what is the relationship between interoceptive awareness and PTSD? The purpose of this scoping review was to ascertain any consensus amongst the literature and identify gaps in the existing literature in order to propose future research projects to enhance investigation in this area.

The data collated in this scoping review displays a significant upturn in studies focusing on interoceptive awareness and PTSD in the past six years. The reasons for this may be two-fold. Firstly, there has been an emergence of a ‘fourth wave’ of psychotherapies which have been thought to make space for alternative and contemporary therapies which promote positive well-being and go beyond symptom recovery (71). Mind-body integration and the mechanism behind the efficacy of therapies promoting a bottom-up approach has become a feature in this fourth wave. This is suggested to have generated interest around interoceptive awareness and its pertinence in psychiatric and behavioural disorders. Secondly, the global and societal events occurring in the past four years has possibly seen an increased focus on understanding the development, maintenance, and treatment of PTSD. The COVID-19 pandemic had, and is still having, a traumatic impact on the mental health of the general public across the world. A comprehensive review of the literature regarding the consequences of the pandemic highlighted particularly significant effects on healthcare workers and COVID-19 survivors with increases in PTSD being documented across the world (72). It is proposed that possible reasons for the significant increase of research into interoceptive awareness and PTSD together is due to the likely connection between interoceptive awareness and emotion regulation, as outlined in this scoping review, and the well-documented association with emotion dysregulation in PTSD. This, coupled with a global pandemic, which placed stress and trauma-related disorders at the forefront of awareness, are possible explanations for this growth in research. The increased focus on this topic has brought about a greater understanding into this field, however, this scoping review revealed complexities around defining and measuring interoceptive awareness in individuals with PTSD worth exploring.

### Defining interoceptive awareness

In relation to the first research question, how is interoceptive awareness defined in the literature on interoceptive awareness and PTSD, the literature revealed a wide variety of terminology used to describe a single construct that overall has consensus around the meaning. Included in the widely accepted global definition is the ability to observe physiological sensations occurring in the viscera which provides an individual with information regarding their physical and emotional state (30). In clinical psychology, the quality of observation and attention that is placed on the physical sensations arising in the body are critical in determining the adaptiveness of this ability and is therefore deemed important to be included in the definition. A more specific definition for this purpose repeatedly emerged in the literature by Mehling et al. (33), p. 2): interoceptive awareness is “the sensory awareness that originates from the body’s physiological states, processes, and actions, and functions as an interactive process that includes a person’s appraisal and is shaped by attitudes, beliefs, and experience in their social and cultural context”.

### Measuring interoceptive awareness

The second research question aimed to understand how interoceptive awareness is measured in the research concerning PTSD. Largely interoceptive awareness as it pertains to PTSD, has been measured in two ways. Either through the use of a subjective self-report questionnaire, such as the MAIA-2 or through neuroimaging to assess the functioning of identified areas of the brain associated with interoceptive awareness. The literature included in this scoping review identified minimal use of objective measurement techniques such as the heartbeat counting task or heartbeat detection test when assessing interoceptive awareness in individuals with PTSD. One reason for this is suggested to be due to the issues that have been raised on the effectiveness of the heartbeat counting task as the only measurement of interoceptive awareness. Murphey et al. (73) have put forward the question as to whether the heartbeat counting task appropriately measures interoceptive awareness due to a lack of studies which appropriately control for confounds which may alter the outcome e.g. knowledge of average resting heart rate and participants being told to guess the number of heartbeats if they cannot recognise them etc. The heartbeat detection test, where individuals are asked to identify if an audio tone, which is sounded with a slight delay after the individual’s heartbeat, occurs synchronously with their heartbeat, has similarly had concerns regarding its efficacy (74). It is suggested that individuals may sense their heartbeat in different locations in their body with delays in their perception occurring if they experience the sensations in peripheral body parts as opposed to central body parts (75). This flags concerns with the use of specific time delays between the tone and the individual’s heartbeat as perception of the heartbeat may therefore appear to occur out of synchrony and lead to inaccurate results in ability (75). Lastly, it has been proposed that objective measures such as the heartbeat counting and heartbeat detection tests measure what has been termed interoceptive accuracy (5) which does not distinguish between the attentional style directed towards the physiological sensations, deemed to be of high clinical importance (76). The concerns regarding the efficacy of the common objective measurements in assessing interoceptive awareness as well as the lack of inclusion of attentional style provides a rationale as to the increased use of self-report measures in this research space.

The multidimensional assessment of interoceptive awareness (MAIA) and MAIA-2 (35) was the most utilised subjective self-report questionnaire in the literature pertaining to interoceptive awareness and PTSD. The high use of this measure in research is proposed to be in part due to the practicality of this measure, 37 self-report items, as well as the sound psychometric properties it boasts. Mehling et al. (35) identified that the MAIA-2 supports an eight-factor model of interoceptive awareness consisting of the following scales: noticing (awareness of body sensations); not-distracting (tendency not to distract from uncomfortable body sensations); not-worrying (tendency not to worry about uncomfortable body sensations); attention regulation (ability to sustain attention to body sensations); emotional awareness (awareness of the association between emotions and body sensations); self-regulation (ability to regulate distress by attending to body sensations); body listening (actively listening to the body for information); and body trusting (ability to experience the body as safe).

### Function of interoceptive awareness

The third research question explored the function of interoceptive awareness. The primary function of interoceptive awareness documented in the data collated in this scoping review was the role it played in an individual’s ability to regulate their emotions. Emotion regulation was proposed to be improved through having a mindful awareness on physical sensations occurring in the body by reducing an individual’s reactivity to these sensations (48). This result can be explained through the theoretical framework outlined by Price & Hooven (17). Interoceptive awareness is suggested to involve a two-way communication (17) whereby messages about the physical state of the body, including the sensations associated with emotions are transmitted to cortical areas (30). From there appropriate responses can be applied to support physical and emotional well-being (30). Emotion regulation requires an awareness and understanding of the emotional sensations arising from the body, in other words, interoceptive awareness (17). Interoceptive awareness provides a way of accurately detecting and evaluating physiological cues, allowing an individual to effectively respond in ways which help support optimal well-being (17). This function is corroborated in the literature outside of that included in this study which exclusively looked at the data involving interoceptive awareness and PTSD. It was proposed that there is a link between interoceptive awareness of physical sensations associated with emotions and the ability to regulate affect and engage in effective decision-making (16, 17). This connection is further supported through studies displaying poorer interoceptive awareness to be present in individuals experiencing emotional disorders (11). Furthermore, greater interoceptive awareness has been identified to enable the downregulation of emotions and an individual’s ability to perceive their internal bodily cues determines their emotion regulation ability (18). Understanding abilities that improve emotion regulation is an important and critical finding as the association between emotion regulation and mental health disorders has been well documented.

### Interoceptive awareness and PTSD

The final research question sought to understand if there was a relationship between interoceptive awareness and PTSD, and if so, what is the nature of this relationship. This scoping review revealed a promising foundation in establishing a positive relationship between interoceptive awareness and PTSD. The literature revealed a combination of neuroimaging studies and clinical intervention trials as being compelling sources of evidence for the existence and nature of the relationship.

Neuroimaging studies in this scoping review were mixed. More studies largely concluded that a combination of structural and functional impairments in areas of the brain reported to be associated with interoceptive awareness, the medial prefrontal cortex and anterior insula, are smaller or experience less activation in individuals with PTSD. This suggests possible impairment in the functioning of these brain regions or the subsequent connectivity of these regions to other regions important for emotion or self-regulation, reducing an individual’s ability to attend to and interpret sensations residing in the body. To further support the relationship between PTSD and interoceptive awareness, neuroimaging studies have investigated brain functioning with individuals who experience dissociation, a specific subtype of PTSD. The results from this scoping review further suggest diminished activation of the insular during times of dissociation which corresponds to individuals reporting difficulties attending to and experiencing physiological sensations. In light of this evidence, Lanius et al. (54) promote restoration of interoceptive awareness in individuals experiencing hypo-arousal as a treatment strategy to regain functioning of the insula.

Despite the emergence of the relationship between interoceptive awareness and PTSD, not all studies have shown a reduction in activation of certain brain areas, but rather an increased activation of the anterior insula, suggesting heightened levels of body awareness or interoception (60, 61). It is unclear if in these studies demonstrating increased activation of the anterior insula, whether there is a consequential heightened ruminative self-focus on physiological symptoms, as opposed to the adaptive mindful appraisal of physical sensations. Additionally, there is a lack of evidence that surfaced during this scoping review which directly displays this association between impairment in specific brain areas equating to loss of interoceptive awareness ability. Therefore, based on the neuroimaging studies available in this scoping review this direct relationship continues to appear somewhat speculative.

Clinical intervention trials have further provided important information regarding the relationship between interoceptive awareness and PTSD. Overall, from the clinical intervention studies in this scoping review, the most poignant outcome relevant was the lack of statistical analysis displaying the mediating effect of interoceptive awareness on PTSD symptomology. Whilst there were studies displaying various interventions to significantly reduce PTSD symptomology and enhance interoceptive awareness in conjunction with one another, no studies examined if the reduction observed in PTSD symptomology was in part due to the improvement in interoceptive awareness. Therefore, whilst a relationship between interoceptive awareness and PTSD can be inferred, it continues to remain speculative without direct statistical analysis. Additionally, another notable outcome was the presence of the interoceptive awareness measure, MAIA and MAIA-2, and the specific elements of interoceptive awareness which appeared to improve and elements which did not improve alongside the reduction of PTSD symptomology. Self-regulation (ability to regulate psychological distress by paying attention to internal sensations) and body listening (listening to the body for insight) were two elements demonstrated in two separate studies to improve with specific interventions, mostly focusing on exercise, which also found a reduction in PTSD symptomology. Whilst it cannot be concluded that the elements of interoceptive awareness, self-regulation and body listening, are responsible for the reduction in PTSD symptomology it does pose a possibility that being attuned to your physiological symptoms learning how to regulate your distress through this may be of benefit for individuals with PTSD symptomology. This aligns with the notion that avoidance of emotional and physical sensations may perpetuate individuals’ level of distress (77). Furthermore, the results of this scoping review also brought light onto the application of the BBAT for individuals with PTSD. The emergence of this therapy in the literature for PTSD indicates the highlighting of the body as a central focal point for treatment. The expected outcome of this therapy is the improvement of interoceptive awareness in the short-term which will have a long-term impact on reducing PTSD (38). It is again important to note that no longitudinal studies have demonstrated evidence that it is directly through the improvement of interoceptive awareness that PTSD symptoms will reduce.

This research demonstrates that individuals with PTSD may experience lower levels of interoceptive awareness, manifesting as difficulty in identifying and interpreting bodily sensations, emotions, and physical states. This in turn is predicted to impact on an individual’s ability to manage PTSD symptomology. To the author’s knowledge, despite there being theoretical and empirical support, there are no studies which directly investigate or model the relationship between interoceptive awareness and PTSD in a clinical sample. Additionally, whether PTSD symptoms are the cause of impaired interoceptive awareness through alterations in the way sensory information in experienced and interpreted by the brain and nervous system, further exacerbating PTSD symptoms, is yet to be explored, leaving a significant gap in the literature and in evidence-based clinical practice.

## Conclusion and future research directions

This scoping review on interoceptive awareness and PTSD revealed a well-supported connection between the two, interlaced with a complexity regarding varying definitions, assessment measures, and a rapidly emerging literature base. The evidence supports the utilisation of a definition of interoceptive awareness to include one that includes the quality of cognitive appraisal and focuses on the adaptive mindful approach to internal physical sensations as opposed to the heightened ruminative self-focus. Additionally, the evidence lends itself to the utilisation of the MAIA-2 as a measure for interoceptive awareness and exploring the breakdown of the elements suggested to be involved in interoceptive awareness and how they relate to PTSD. Furthermore, there are many theories and models that highlight a relationship between interoceptive awareness, emotion regulation, and PTSD, however, a notable gap is evident in these models being tested in a clinical population of individuals experiencing PTSD. Lastly, future research should aim to incorporate a wider range of terminology that were not incorporated into this scoping review e.g., interoceptive inferences to continue enhancing our knowledge into this area.

## Data availability statement

The data analyzed in this study is subject to the following licenses/restrictions. This is a review paper only - of existing publications. Requests to access these datasets should be directed to kleech@bond.edu.au.

## Author contributions

KL: Conceptualization, Methodology, Writing – review & editing, Formal analysis, Writing – original draft. PS: Conceptualization, Methodology, Writing – review & editing, Supervision. AP: Supervision, Writing – review & editing.

## References

[1] Sherrington C . The integrative action of the nervous system. New Haven, CT: Yale University Press (1906).

[2] Cameron OG . Interoception: The inside story—A model for psychosomatic processes. Psychosomatic Med. (2001) 63:697–710. doi: 10.1097/00006842-200109000-00001 11573016

[3] Schulz A Vögele C . Interoception and stress. Front Psychol. (2015) 6:993. doi: 10.3389/fpsyg.2015.00993 26257668 PMC4507149

[4] Gibson JL . ACEs wild: making meaning out of trauma through altruism born of suffering. New England: Antioch University (2019).

[5] Garfinkel SN Seth AK Barrett AB Suzuki K Critchley HD . Knowing your own heart: Distinguishing interoceptive accuracy from interoceptive awareness. Biol Psychol. (2015) 104:65–74. doi: 10.1016/j.biopsycho.2014.11.004 25451381

[6] Murphy J Catmur C Bird G . Classifying individual differences in interoception: Implications for the measurement of interoceptive awareness. Psychonomic Bull Rev. (2019) 26:1467–71. doi: 10.3758/s13423-019-01632-7 PMC6797703 31270764

[7] Pavlov IP . Conditioned reflexes; an investigation of the physiological activity of the cerebral cortex. London: Oxford University Press (1927). 10.5214/ans.0972-7531.1017309 PMC4116985 25205891

[8] Ceunen E Vlaeyen JW Van Diest I . On the origin of interoception. Front Psychol. (2016) 7:743. doi: 10.3389/fpsyg.2016.00743 27242642 PMC4876111

[9] Razran G . The observable and the inferable conscious in current Soviet psychophysiology: Interoceptive conditioning, semantic conditioning, and the orienting reflex. psychol Rev. (1961) 68:68–81. doi: 10.1037/h0039848 13740033

[10] Pavlov IP . Lectures on conditioned reflexes. Vol. II. Conditioned reflexes and psychiatry. Arch Neurol Psychiatry. (1941) 49:637. doi: 10.1001/archneurpsyc.1943.02290160159017

[11] Paulus MP Stein MB . Interoception in anxiety and depression. Brain Structure Funct. (2010) 214:451–63. doi: 10.1007/s00429-010-0258-9 PMC2886901 20490545

[12] Davidson TL . The nature and function of interoceptive signals to feed: toward integration of physiological and learning perspectives. psychol Rev. (1993) 100:640–57. doi: 10.1037/0033-295X.100.4.640 8255952

[13] Paulus MP Tapert SF Schulteis G . The role of interoception and alliesthesia in addiction. Pharmacol Biochem Behav. (2009) 94:1–7. doi: 10.1016/j.pbb.2009.08.005 19698739 PMC2753707

[14] Rothschild B . The body remembers continuing education test: The psychophysiology of trauma & trauma treatment. New York: WW Norton & Company (2000).

[15] Craig AD . (2015). Princeton, NJ: Princeton University Press.

[16] Dunn BD Galton HC Morgan R Evans D Oliver C Meyer M . Listening to your heart. psychol Sci. (2010) 21:1835–44. doi: 10.1177/0956797610389191 21106893

[17] Price CJ Hooven C . Interoceptive awareness skills for emotion regulation: Theory and approach of mindful awareness in body-oriented therapy (MABT). Front Psychol. (2018) 9:798=. doi: 10.3389/fpsyg.2018.00798= 29892247 PMC5985305

[18] Füstös J Gramann K Herbert BM Pollatos O . On the embodiment of emotion regulation: Interoceptive awareness facilitates reappraisal. Soc Cogn Affect Neurosci. (2013) 8:911–7. doi: 10.1093/scan/nss089 PMC3831556 22933520

[19] Werner NS Schweitzer N Meindl T Duschek S Kambeitz J Schandry R . Interoceptive awareness moderates neural activity during decision-making. Biol Psychol. (2013) 94:498–506. doi: 10.1016/j.biopsycho.2013.09.002 24076035

[20] Furman DJ Waugh CE Bhattacharjee K Thompson RJ Gotlib IH . Interoceptive awareness, positive affect, and decision making in major depressive disorder. J Affect Disord. (2013) 151:780–5. doi: 10.1016/j.jad.2013.06.044 PMC3797260 23972662

[21] Van der Kolk BA . Clinical implications of neuroscience research in PTSD. Ann New York Acad Sci. (2006) 1071:277–93. doi: 10.1196/annals.1364.022 16891578

[22] Yehuda R . Biology of posttraumatic stress disorder. J Clin Psychiatry. (2000) 61:14–21. 10795605

[23] Levine PA . Accumulated stress, reserve capacity and disease. Ann Arbor, MI: University of California, Berkeley (1977).

[24] Levine PA . Waking the tiger: Healing trauma: The innate capacity to transform overwhelming experiences. Berkeley, CA: North Atlantic Books (1997).

[25] Levine PA . In an unspoken voice: how the body releases trauma and restores goodness. Berkeley, CA: North Atlantic Books (2010).

[26] Payne P Levine PA Crane-Godreau MA . Somatic experiencing: Using interoception and proprioception as core elements of trauma therapy. Front Psychol. (2015) , 6:93. doi: 10.3389/fpsyg.2015.00093 25699005 PMC4316402

[27] Ogden P Minton K Pain C . Trauma and the body: A sensorimotor approach to psychotherapy. New York: W. W. Norton & Company (2006).

[28] Munn Z Peters MD Stern C Tufanaru C McArthur A Aromataris E . Systematic review or scoping review? Guidance for authors when choosing between a systematic or scoping review approach. BMC Med Res Method. (2018) 18:1–7. doi: 10.1186/s12874-018-0611-x PMC6245623 30453902

[29] Arksey H O’Malley L . Scoping studies: Towards a methodological framework. Int J Soc Res Methodology: Theory Pract. (2005) 8:19–32. doi: 10.1080/1364557032000119616

[30] Craig AD . Interoception: The sense of the physiological condition of the body. Current Opinion in Neurobiology (2003) 13:500–5. doi: 10.1016/S0959-4388(03)00090-4 12965300

[31] Mehling WE Price C Daubenmier JJ Acree M Bartmess E Stewart A . The multidimensional assessment of interoceptive awareness (MAIA). PloS One. (2012) 7:e48230. doi: 10.1371/journal.pone.0048230 23133619 PMC3486814

[32] Reinhardt KM Zerubavel N Young AS Gallo M Ramakrishnan N Henry A . A multi-method assessment of interoception among sexual trauma survivors. Physiol Behav. (2020) 226:113108. doi: 10.1016/j.physbeh.2020.113108 32721494

[33] Mehling WE Gopisetty V Daubenmier J Price CJ Hecht FM Stewart A . Body awareness: Construct and self-report measures. PloS One. (2009) 4:e5614. doi: 10.1371/journal.pone.0005614 19440300 PMC2680990

[34] Silverberg R . Trauma center trauma-sensitive yoga (TC-TSY) peer support groups: An adjunct modality in a feminist approach to trauma treatment for survivors of sexual violence. In: Dissertation abstracts international: section B: the sciences and engineering, vol. 81. (2020). Available at: http://ovidsp.ovid.com/ovidweb.cgi?T=JS&PAGE=reference&D=psyc17&NEWS=N&AN=2020-17191-034.

[35] Mehling WE Acree M Stewart A Silas J Jones A . The multidimensional assessment of interoceptive awareness, version 2 (MAIA-2). PloS One. (2018) 13:e0208034. doi: 10.1371/journal.pone.0208034 30513087 PMC6279042

[36] Dieterich-Hartwell R . Dance/movement therapy in the treatment of post traumatic stress: A reference model. Arts Psychother. (2017) 54:38–46. doi: 10.1016/j.aip.2017.02.010

[37] Drapkin J . Integrating yoga and self-psychology: An open-trial pilot study. In: Dissertation Abstracts International: Section B: The Sciences and Engineering (2019). p. 80(6–B(E)). Available at: http://ovidsp.ovid.com/ovidweb.cgi?T=JS&PAGE=reference&D=psyc16&NEWS=N&AN=2019-41128-112.

[38] Ahlmark NG Dahl A Andersen HS Tjørnhøj-Thomsen T Andersen S . Body therapy versus treatment as usual among Danish veterans with PTSD: Study protocol for a randomised controlled trial combined with a qualitative study. Contemp Clin Trials Communications,19. (2020) 100596. doi: 10.1016/j.conctc.2020.100596 PMC7322676 32617435

[39] Davis LW Schmid AA Daggy JK Yang Z O’Connor CE Schalk N . Symptoms improve after a yoga program designed for PTSD in a randomized controlled trial with veterans and civilians. In: Psychological Trauma: Theory, Research, Practice, and Policy. Advance online publication (2020). doi: 10.1037/tra0000564 32309986

[40] Hodgdon HB Anderson FG Southwell E Hrubec W Schwartz R . Internal family systems (IFS) therapy for posttraumatic stress disorder (PTSD) among survivors of multiple childhood trauma: A pilot effectiveness study. J Aggression Maltreatment Trauma. (2022) 31:22–43. doi: 10.1080/10926771.2021.2013375

[41] Smith AR Dodd DR Ortiz S Forrest LN Witte TK . Interoceptive deficits differentiate suicide groups and associate with self-injurious thoughts and behaviors in a military sample. Suicide Life-Threatening Behav. (2020) 50:472–89. doi: 10.1111/sltb.12603 31743463

[42] van Wingen GA Geuze E Vermetten E Fernandez G . Perceived threat predicts the neural sequelae of combat stress. Mol Psychiatry. (2011) 16:664–71. doi: 10.1038/mp.2010.132 PMC3100568 21242990

[43] Mitchell KS Wells SY Mendes A Resick PA . Treatment improves symptoms shared by PTSD and disordered eating. J Traumatic Stress. (2012) 25:535–42. doi: 10.1002/jts.21737 23073973

[44] Nicholson AA Sapru I Densmore M Frewen PA Neufeld RW Théberge J . Unique insula subregion resting-state functional connectivity with amygdala complexes in posttraumatic stress disorder and its dissociative subtype. Psychiatry Research: Neuroimaging. (2016) 250:61–72. doi: 10.1016/j.pscychresns.2016.02.002 27042977

[45] Simmons A Strigo IA Matthews SC Paulus MP Stein MB . Initial evidence of a failure to activate right anterior insula during affective set shifting in Posttraumatic Stress Disorder. Psychosomatic Med. (2009) 71:373–7. doi: 10.1097/PSY.0b013e3181a56ed8 PMC2888032 19398499

[46] Calì G Ambrosini E Picconi L Mehling W Committeri G . Investigating the relationship between interoceptive accuracy, interoceptive awareness, and emotional susceptibility. Front Psychol. (2015) 61202:1202. doi: 10.3389/fpsyg.2015.01202 PMC4547010 26379571

[47] McFarland RA . Heart rate perception and heart rate controll. Psychophysiology. (1975) 12(4):402–5. doi: 110.1111/j.1469-8986.1975.tb00011.x 1162006 10.1111/j.1469-8986.1975.tb00011.x

[48] Weng HY Feldman JL Leggio L Napadow V Park J Price CJ . Interventions and manipulations of interoception. Trends Neurosci. (2021) 44:52–62. doi: 10.1016/j.tins.2020.09.010 33378657 PMC7805576

[49] Herbert BM Herbert C Pollatos O . On the relationship between interoceptive awareness and alexithymia: Is interoceptive awareness related to emotional awareness? J Pers. (2011) 79:1149–75. doi: 10.1111/jopy.2011.79.issue-5 21241306

[50] Schimmenti A Caretti V . Linking the overwhelming with the unbearable: Developmental trauma, dissociation, and the disconnected self. Psychoanalytic Psychol. (2016) 33:106–28. doi: 10.1037/a0038019

[51] Monteleone AM Mereu A Cascino G Criscuolo M Castiglioni MC Pellegrino F . Re-conceptualization of anorexia nervosa psychopathology: A network analysis study in adolescents with short duration of the illness. Int J Eating Disord. (2019) 52:1263–73. doi: 10.1002/eat.23137 31313374

[52] Boettcher H Brake CA Barlow DH . Origins and outlook of interoceptive exposure. J Behav Ther Exp Psychiatry. (2016) 53:41–51. doi: 10.1016/j.jbtep.2015.10.009 26596849

[53] Wolpe J . Psychotherapy by reciprocal inhibition. California: Stanford University Press (1958).

[54] Lanius RA Frewen PA Tursich M Jetly R McKinnon MC . Restoring large-scale brain networks in PTSD and related disorders: A proposal for neuroscientifically-informed treatment interventions. Eur J Psychotraumatol. (2015) 627313. doi: 10.3402/ejpt.v6.27313 PMC4390556 25854674

[55] Markowitsch HJ Kessler J Weber-Luxenburger G van der Ven C Albers M Heiss WD . Neuroimaging and behavioral correlates of recovery from mnestic block syndrome and other cognitive deteriorations. . Neuropsychiatry Neuropsychology Behav Neurol. (2000) 13:60–6. 10645738

[56] Shin LM Rauch SL Pitman RK . Amygdala, medial prefrontal cortex, and hippocampal function in PTSD. Ann New York Acad Sci. (2006) 1071:67–79. doi: 10.1196/annals.1364.007 16891563

[57] Morgan MA Romanski LM LeDoux JE . Extinction of emotional learning: Contribution of medial prefrontal cortex. Neurosci Lett. (1993) 163:109–13. doi: 10.1016/0304-3940(93)90241-C 8295722

[58] Vasterling JJ Brailey K Constans JI Sutker PB . Attention and memory dysfunction in posttraumatic stress disorder. Neuropsychology. (1998) 12:125–33. doi: 10.1037/0894-4105.12.1.125 9460740

[59] Craig AD . How do you feel? Interoception: The sense of the physiological condition of the body. Nat Rev Neurosci. (2002) 3:655–66. doi: 10.1038/nrn894 12154366

[60] Paulus MP Stein MB . An insular view of anxiety. Biol Psychiatry. (2006) 60:383–7. doi: 10.1016/j.biopsych.2006.03.042 16780813

[61] Hopper JW Frewen PA van der Kolk BA Lanius RA . Neural correlates of reexperiencing, avoidance, and dissociation in PTSD: Symptom dimensions and emotion dysregulation in responses to script-driven trauma imagery. J Traumatic Stress. (2007) 20:713–25. doi: 10.1002/jts.20284 17955540

[62] Cross D Fani N Powers A Bradley B . Neurobiological development in the context of childhood trauma. Clin Psychology: Sci Pract. (2017) 24:111–24. doi: 10.1111/cpsp.12198 PMC6428430 30906116

[63] Fetzner MG Asmundson GJG . Aerobic exercise reduces symptoms of posttraumatic stress disorder: A randomized controlled trial. Cogn Behav Ther. (2015) 44:301–13. doi: 10.1080/16506073.2014.916745 24911173

[64] Schwartz R . Introduction to the internal family systems model. Oak Park, IL: Trailhead Publications (2001).

[65] Elton J Stage K Sternhagen Nielsen AB Hjort Svendsen AL . The experience of Basic Body Awareness Therapy and its transfer to daily life amongst Danish military veterans with PTSD. *Journal of* . Bodywork Movement Therapies. (2021) 28:202–11. doi: 10.1016/j.jbmt.2021.07.001 34776142

[66] Andersen MR Clausen A Sternhagen Nielsen AB Hjort Svendsen AL . Experiences with basic body awareness therapy as an add-on to cognitive behavioural therapy among Danish military veterans with PTSD: An interview study. J Bodywork Movement Therapies. (2021) 27:550–9. doi: 10.1016/j.jbmt.2021.03.023 34391286

[67] Williston SK Grossman D Mori DL Niles BL . Mindfulness interventions in the treatment of posttraumatic stress disorder. Prof Psychology: Res Pract. (2021) 52:46–57. doi: 10.1037/pro0000363

[68] Farb N Daubenmier J Price CJ Gard T Kerr C Dunn BD . Interoception, contemplative practice, and health. Front Psychol. (2015) 6:763. doi: 10.3389/fpsyg.2015.00763 26106345 PMC4460802

[69] Van der Kolk BA . The body keeps the score. Viking: New York (2014).

[70] Vierling V Etori S Valenti L Lesage M Pigeyre M Dodin V . Prévalence et impact de l’état de stress post-traumatique chez les patients atteints de troubles du comportement alimentaire [Prevalence and impact of post-traumatic stress disorder in a disordered eating population sample]. Presse Med. (2015) 44:341–52. doi: 10.1016/j.lpm.2015.04.039 26433833

[71] Peteet JR . A fourth wave of psychotherapies: Moving beyond recovery toward well-being. Harvard Rev Psychiatry. (2018) 26:90–5. doi: 10.1097/HRP.0000000000000155 29394174

[72] Chamaa F Bahmad HF Darwish B Kobeissi JM Hoballah M Nassif SB . PTSD in the COVID-19 era. Curr Neuropharmacology. (2021) 19:2164–79. doi: 10.2174/1570159X19666210113152954 PMC9185760 33441072

[73] Murphy J Brewer R Hobson H Catmur C Bird G . Is alexithymia characterised by impaired interoception? Further evidence, the importance of control variables, and the problems with the Heartbeat Counting Task. Biol Psychol. (2018) 136:189–97. doi: 10.1016/j.biopsycho.2018.05.010 29803614

[74] Brener J Kluvitse C . Heartbeat detection: Judgments of the simultaneity of external stimuli and heartbeats. Psychophysiology. (1988) 25:554–61. doi: 10.1111/j.1469-8986.1988.tb01891.x 3186884

[75] Plans D Ponzo S Morelli D Cairo M Ring C Keating CT . Measuring interoception: The phase adjustment task. Biol Psychol. (2021) 165:108171. doi: 10.1016/j.biopsycho.2021.108171 34411620

[76] Mehling W . Differentiating attention styles and regulatory aspects of self-reported interoceptive sensibility. Philos Trans R Soc B: Biol Sci. (2016) 371:20160013. doi: 10.1098/rstb.2016.0013 PMC5062101 28080970

[77] Spinhoven P Drost J de Rooij M van Hemert AM Penninx BW . A longitudinal study of experiential avoidance in emotional disorders. Behav Ther. (2014) 45:840–50. doi: 10.1016/j.beth.2014.07.001 25311292

